# Supplementary material for: Structural basis of reactivation of oncogenic p53 mutants by a small molecule: methylene quinuclidinone (MQ)
Source: Nat Commun. 2021 Dec 3;12:7057. doi: 10.1038/s41467-021-27142-6 (PMC8642532; doi:10.1038/s41467-021-27142-6)
Supplement: Supplementary file 1 — Supplementary Information [file 41467_2021_27142_MOESM1_ESM.pdf]

Supplementary Information

Structural basis of reactivation of oncogenic p53 mutants by a small molecule: methylene quinuclidinone (MQ)

Oksana Degtjarik[1], Dmitrij Golovenko[1], Yael Diskin-Posner[2], Lars Abrahmsén[3], Haim Rozenberg[1] and Zippora Shakked[1]

[1]Department of Structural Biology and [2]Department of Chemical Research Support, Weizmann Institute of Science, 76100 Rehovot, Israel

[3]Aprea Therapeutics AB, 17165 Solna, Sweden

Content

Supplementary Note 1. 2D chemical schemes of MQ and the modified residues

Supplementary Table 1. Crystallization and soaking conditions

Supplementary Table 2. X-ray data collection and statistics of R273H-related structures

Supplementary Table 3. X-ray data collection and statistics of R273C-related structures

Supplementary Table 4. X-ray data collection and statistics of R282W-related structures

Supplementary Table 5. X-ray data collection and statistics of wt-DNA-MQ structures

Supplementary Table 6. Refinement statistics of R273H-related structures

Supplementary Table 7. Refinement statistics of R273C-related structures

Supplementary Table 8. Refinement statistics of R282W-related structures

Supplementary Table 9. Refinement statistics of wt-DNA-MQ structures

Supplementary Table 10. Schematic presentations of the DNA response elements of the p53-DNA complexes

Supplementary Table 11. Unresolved residues in L1 loop (113-124)

Supplementary Fig. 1. Divergent stereo view of superposed two conformations of L2 loops at C182 residues in the presence and absence of bound MQ

Supplementary Fig. 2. Comparison of MQ-modified p53 monomers from R273H-MQ and R273C-MQ structures with the structure of wild-type p53DBD bound to DNA

Supplementary Fig. 3. Compatibility of MQ-C273 conjugates with DNA binding

Supplementary Fig. 4. Overall views of MQ-Cys conjugates within their core domains observed in p53-DNA-MQ structures

Supplementary Fig. 5. Close up views of the accessible space around C124 residues in free and DNA-bound proteins

Supplementary Fig. 6. Stereo views of local conformational changes in p53-DNA structures caused by replacing R282 by W282 and by MQ binding to R282W-DNA

Supplementary Fig. 7. A stereo view of MQ bound to K101 in wt-DNA-MQ (P1) structure

Supplementary Fig. 8. A close-up stereo view of the interactions of two symmetry-related MQ-C277 conjugates with the central DNA helix in R273H-DNA-MQ structure

Supplementary Fig. 9. MQ-Cys conjugates and nearby amino acids and nucleotides shown within their electron density maps (2mF_o-DF_c contoured at 1.0 σ level)

Supplementary Discussion

Supplementary Note 1.

The Michael addition reaction of MQ, 2-methylene-quinuclidin-3-one, results in the formation of covalently-bound enantiomers MQr and MQs. MQ is a prochiral molecule, acting as a Michael donor by forming chiral centers with thiol or with amino groups (displayed in the chemical diagrams below).

Due to technical limitations of the validation process at the Protein Data Bank (wwPDB), the enantiomers MQr and MQs, covalently bound to amino acids in our structures and referred as QNN and QN8 in the relevant entries, were described by default as fully-protonated free molecules. The free QNN and QN8 enantiomers are two artificial entities not observed in our structures. The chiral definitions of QNN and QN8 bound to cysteines is reversed to that of the artificial free ligands.

2D chemical schemes of MQ and the modified residues

Acronym	MQ
Name	Methylene quinuclidinone, 2-methylene-quinuclidin-3-one
IUPAC	2-methylidene-1-azabicyclo[2.2.2]octan-3-one
Formula	C8 H11 N O
SMILES	C=C1C(=O)C2CCN1CC2

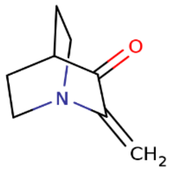

Acronym	MQr-Cys
Name	S-[(2R)-3-oxoquinuclidin-2-yl]methyl-L-cysteine
Formula	C11 H18 N2 O3 S
SMILES	O=C1[C@@H](N2CCC1CC2)CSC[C@H](N)C(=O)O

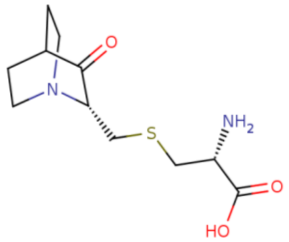

Acronym	MQs-Cys
Name	S-[(2S)-3-oxoquinuclidin-2-yl]methyl-L-cysteine
Formula	C11 H18 N2 O3 S
SMILES	O=C1[C@H](N2CCC1CC2)CSC[C@H](N)C(=O)O

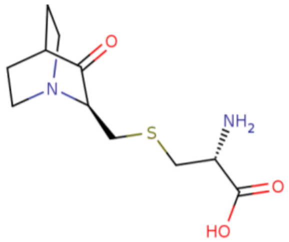

Acronym	MQr-Lys
Name	N6-[(2R)-3-oxoquinuclidin-2-yl]methyl-L-lysine
Formula	C14 H25 N3 O3
SMILES	O=C1[C@H](N2CCC1CC2)CNCCCC[C@H](N)C(=O)O

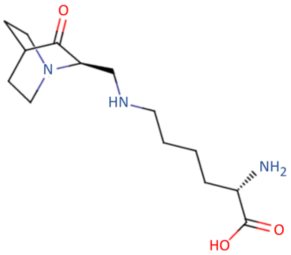

Supplementary Table 1. Crystallization and soaking conditions

Crystals	Protein concentration[a] (mg/ml)	Protein/DNA duplex ratio[b]	MQ Co-crystallization/ Soaking			Reservoir solution	
			Method	Concentration (mM)	Time (hh:mm)	Buffer and salt	PEG 3350 (% w/v)
**R273H**							
**R273H-MQ (I)[c]**	2.5		SO[d]	25	1:00	0.1M Lithium acetate	16
**R273H-MQ (II)**	2.5		CO	50		0.2M Lithium acetate, 2.0% Agarose	20
**R273H-DNA-MQ[e]**	1.9	1:1.4	CO-SO	2/25[f]	1:00	Initial: 0.1M Sodium formate<br>Host drop:0.075M Sodium formate	10<br>7.5
**R273H-DNA[e]**	1.9	1:1.4	CO	2		Initial: 0.1M Sodium formate<br>Host drop:0.075M Sodium formate	10<br>7.5
**R273C**							
**R273C-MQ (I)[g]**	2.15	1:2.4	CO-SO	12/20[f]	0:40	0.2M Sodium fluoride	20
**R273C-MQ (II)**	2.15		CO	20		0.2M Sodium fluoride	20
**R273C/S240R-DNA-MQ**	2.25	1:2.4	SO	25	2:30	0.2M Sodium formate	20
**R282W**							
**R282W-DNA-MQ**	1.9	1:1.5	SO	25	1:00	0.1M Sodium aceteate	20
**R282W-DNA (I)**	2.15	1:1.5				0.03M Citric acid, 0.07M BIS-TRIS propane pH=7.6	20
**R282W-DNA (II)**	2.4	1:1.5	CO	0.5		0.2M Sodium acetate	20
**WT**							
**wt-DNA-MQ (I)**	2.2	1:2.4	SO	25	12:00	0.1M TRIS pH=8.5, 2.0% Tacsimate[tm] pH=8.0	16
**wt-DNA-MQ (II)**	2.5	1:2.4	SO	50	1:30	0.1M TRIS pH=8.5, 2.0% Tacsimate[tm] pH=8.0	16
**wt-DNA-MQ (III)**	2.2	1:2.4	SO	50	3:15	0.1M TRIS pH=8.5, 2.0% Tacsimate[tm] pH=8.0	16
**wt-DNA-MQ (P1)**	2.5	1:2.4	SO	20	1:30	0.1M DL-Malic acid pH=7.0	19

[a] Protein concentration in the 4μl crystallization drop.
[b] Protein monomer to DNA duplex ratio.
[c] The crystal was cross-linked using glutaraldehyde (GA).
[d] "SO" stands for soaking, "CO" stands for co-crystallization, "CO-SO" stands for co-crystallization followed by soaking.
[e] Sizable crystals of R273H-DNA were obtained after four rounds of macro-seeding in the presence of MQ. Some of these co-crystals were further soaked in solution containing MQ to obtain R273H-DNA-MQ crystals.
[f] MQ concentrations used for co-crystallization and soaking, respectively.
[g] The free protein crystal was obtained during an attempt to crystallize the protein-DNA complex.

Supplementary Table 2. X-ray data collection and statistics of R273H-related structures

Structure	**R273H-MQ (I)**	**R273H-MQ (II)**	**R273H-DNA-MQ**	**R273H-DNA**
PDB code	7B47	7B48	7B49	7B4A
ESRF beam line	ID23-1	ID23-2	ID23-1	ID30A-3
Detector	Pilatus6M	Pilatus3 2M	Pilatus6M	Eiger X 4M
Wavelength (Å)	0.97200	0.87290	0.97200	0.87290
Space group	$P2_1$		$C2$	
Mosaicity (°)	0.25-0.48	0.42-0.95	0.31-0.50	0.38-0.58
Unit cell (Å,°)	a=69.0<br>b=71.0<br>c=85.1<br>β=90.3	a=69.2<br>b=72.0<br>c=85.2<br>β=90.1	a=136.7<br>b=50.1<br>c=68.4<br>β=93.6	a=136.8<br>b=50.3<br>c=67.5<br>β=92.7
Volume (Å^3)	417115.9	424980.8	466765.4	464181.9
Resolution (Å)	50-1.80	39-2.05	39-1.38	45-1.90
Upper resolution shell (Å)	1.83-1.80	2.09-2.05	1.40-1.38	1.93-1.90
Measured reflections	300,285	153,772	510,562	283,631
Unique reflections $I > 0$	75,959 (3,802)[a]	49,698 (2,127)	93,002 (4,616)	35,603 (1,781)
Completeness (%)	99.1 (99.2)	94.2 (81.1)	98.0 (97.8)	98.2 (98.5)
$< I/\sigma(I) >$	19.2 (2.7)	10.2 (3.2)	13.7 (2.5)	15.5 (3.4)
R_{sym} (I) (%)	6.5 (49.8)	11.4 (25.4)	11.9 (61.8)	13.6 (68.2)
$CC_{1/2}$ (%)	(89.6)	98.6 (91.0)	99.6 (58.7)	99.5 (93.4)
Twin law/twin fraction (%)		-H,-K,L / 0.243		

[a] Values in parentheses refer to the highest-resolution shell.

**Supplementary Table 3. X-ray data collection and statistics of R273C-related structures**			
Structure	**R273C-MQ (I)**	**R273C-MQ (II)**	**R273C/S240R-DNA-MQ**
PDB code	7B4B	7B4C	7B4D
ESRF beam line	ID23-1		Rigaku MicroMax-007HF VariMax Optics
Detector	Pilatus6M		R-AXIS IV++
Wavelength (Å)	0.97200		1.54178
Space group	$P2_1$		$C2$
Mosaicity (°)	0.20-0.69	0.19-0.91	0.45-0.60
Unit cell (Å,°)	a=68.7<br>b=70.7<br>c=85.1<br>β=90.3	a=68.9<br>b=71.1<br>c=84.9<br>β=90.0	a =137.6<br>b=50.2<br>c=34.2<br>β=92.6
Volume ($Å^3$)	413466.2	415805.5	236013.5
Resolution (Å)	40-1.76	50-1.71	25-1.85
Upper resolution shell (Å)	1.79-1.76	1.74-1.71	1.88-1.85
Measured reflections	327,275	524,731	181,291
Unique reflections I> 0	78,718 (3,842)[a]	87,231 (4,326)	19,150 (885)
Completeness (%)	96.8 (95.5)	98.4 (97.0)	95.6 (88.1)
$< I/\sigma(I)>$	13.2 (2.4)	20.1 (2.2)	35.8 (2.7)
R_{sym} (I) (%)	11.3 (57.4)	8.2 (62.6)	5.7 (50.8)
$CC_{1/2}$ (%)	(83.3)	(82.3)	(90.9)
Twin law/twin fraction (%)		-H,-K,L / 0.354	

[a] Values in parentheses refer to the highest-resolution shell.

Supplementary Table 4. X-ray data collection and statistics of R282W-related structures

Structure	**R282W-DNA-MQ**	**R282W-DNA (I)**	**R282W-DNA (II)**
PDB code	7B4E	7B4F	7B4G
ESRF beam line	ID30A-3	Rigaku MicroMax-007HF VariMax Optics	
Detector	Eiger X 4M	R-AXIS IV++	
Wavelength (Å)	0.96770	1.54178	
Space group		*C*2	
Mosaicity (°)	0.31-0.83	0.61-0.88	0.60-0.72
Unit cell (Å,°)	a=136.5<br>b=49.6<br>c=33.4<br>β=93.4	a=137.2<br>b=49.7<br>c=33.8<br>β=93.4	a=137.4<br>b=49.6<br>c=34.1<br>β=93.7
Volume ($Å^3$)	224965.0	230402.7	232839.4
Resolution (Å)	50-1.58	21-1.78	40-1.86
Upper resolution shell (Å)	1.61-1.58	1.81-1.78	1.89-1.86
Measured reflections	306,320	100,983	66,152
Unique reflections $I > 0$	30,188 (1,443)[a]	21,071 (961)	18,811 (922)
Completeness (%)	97.5 (93.5)	95.9 (92.3)	96.2 (91.9)
$< I/\sigma(I) >$	20.4 (2.0)	20.6 (3.1)	13.6 (2.2)
R_{sym} (*I*) (%)	11.1 (61.0)	6.7 (45.2)	8.8 (48.2)
$CC_{1/2}$ (%)	99.6 (71.6)	100 (87.7)	(81.8)

[a] Values in parentheses refer to the highest-resolution shell.

Supplementary Table 5. X-ray data collection and statistics of wt-DNA-MQ structures				
Structure	**wt-DNA-MQ (I)**	**wt-DNA-MQ (II)**	**wt-DNA-MQ (III)**	**wt-DNA-MQ (P1)**
PDB code	6ZNC	7B4N	7B4H	7B46
ESRF beam line	ID29	ID23-1		ID23-1
Detector	Pilatus6M	Pilatus6M		Pilatus6M
Wavelength (Å)	0.97625	0.97600		0.97200
Space group	*C*2			*P*1
Mosaicity (°)	0.45-0.66	0.20-0.29	0.19-0.82	0.16-0.34
Unit cell (Å,°)	a=137.7<br>b=49.5<br>c=34.1<br><br>β=92.8	a=137.8<br>b=50.0<br>c=34.0<br><br>β=93.2	a=137.6<br>b=50.1<br>c=34.1<br><br>β=93.0	a=54.9<br>b=58.3<br>c=78.1<br>α=82.9<br>β=87.5<br>γ=72.8
Volume ($Å^3$)	232165.6	233928.7	234716.9	237551.2
Resolution (Å)	50-1.65	100-1.32	49-1.39	50-2.02
Upper resolution shell (Å)	1.68-1.65	1.34-1.32	1.41-1.39	2.05-2.02
Measured reflections	167,818	575,464	339,541	188,390
Unique reflections $I > 0$	27,447 (1,339)[a]	53,678 (1,730)	46,808 (2,318)	58,675 (2,893)
Completeness (%)	98.4 (98.3)	99.3 (97.5)	99.7 (99.2)	95.5 (94.1)
$\langle I/\sigma(I) \rangle$	22.9 (3.0)	34.9 (3.0)	20.5 (3.0)	14.0 (3.6)
R_{sym} (*I*) (%)	7.0 (51.5)	6.0 (52.4)	9.3 (55.9)	8.2 (41.3)
$CC_{1/2}$ (%)	(88.0)	(89.5)	(85.7)	(89.6)

[a] Values in parentheses refer to the highest-resolution shell.

Supplementary Table 6. Refinement statistics of R273H-related structures

Structure	**R273H-MQ (I)**	**R273H-MQ (II)**	**R273H-DNA-MQ**	**R273H-DNA**
PDB code	7B47	7B48	7B49	7B4A
Resolution range (Å)	49.5-1.8	36.7-2.05	34.1-1.42	31.1-1.9
No. of reflections (*I*> 0)	76,098	49,685	85,297	35,414
No. of reflections in test set	1,996	2,400	4,265	1,766
No. of molecules in the a.u.[a]	4	4	2/0.5	2/0.5
R_{work} (%) / R_{free} (%)	17.8/20.2	17.6/22.8	18.4/22.9	20.7/25.4
No. of TLS groups	31	48	0	23
No. iso/aniso B-factors[b]	6737/4	6615/0	1145/3292	3999/2
No. of protein / DNA atoms	6211/-	6061/-	3212/574	3072/410
No. of solvent atoms[c]	430	534	611	519
No. of MQ conjugates (atoms)	10 (100)	2 (20)	4 (40)	-
<*B* factors> (Å^2)				
- Overall	39.6	31.0	21.8	25.3
- Protein / DNA / Solvent	39.5/ - /39.7	33.7/ - /33.2	16.9/39.8/29.9	23.3/24.4/37.6
- MQ conjugates	48.6	44.3	36.1	-
Root mean square deviations:				
- Bond length (Å) / Angle (°)	0.005/0.90	0.017/2.150	0.008/1.04	0.006/0.82
Ramachandran Plot:				
- Favored (%)	99.0	96.7	100	98.9
- Outliers (%)	0.0	0.40	0.0	0.0
Rotamer outliers (%)	2.0	2.19	2.50	1.48
Twin operator and fraction		-H,-K,L/0.244		

[a] Protein monomers/DNA duplexes
[b] Number of isotropic / anisotropic B-factors
[c] Number of atoms not belonging to protein, to DNA and to bound MQ.

**Supplementary Table 7. Refinement statistics of R273C-related structures**			
Structure	**R273C-MQ (I)**	**R273C-MQ (II)**	**R273C/S240R-DNA-MQ**
PDB code	7B4B	7B4C	7B4D
Resolution range (Å)	36.5-1.76	42.7-1.71	18.3-1.85
No. of reflections (I> 0)	78,674	87,210	19,144
No. of reflections in test set	2,008	4,332	1,922
No. of molecules in the a.u.[a]	4	4	1/0.5
R_{work} (%) / R_{free} (%)	18.0/21.8	14.9/18.2	16.8/20.4
No. of TLS groups	27	40	10
No. iso/aniso B-factors[b]	7035/4	6995/-	2056/1
No. of protein / DNA atoms	6238/-	6343/-	1639/218
No. of solvent atoms[c]	681	582	160
No. of MQ conjugates (atoms)	12 (120)	7 (70)	4 (40)
<*B* factors> ($Å^2$)			
- Overall	31.6	33.0	38.8
- Protein / DNA / Solvent	31.0/-/35.1	32.7/-/35.0	36.4/55.6/38.9
- MQ conjugates	41.1	44.6	42.0
Root mean square deviations:			
- Bond length (Å) / Angle (°)	0.007/0.85	0.020/1.970	0.006/1.24
Ramachandran Plot:			
- Favored (%)	99.46	99.1	100
- Outliers (%)	0.0	0.0	0.0
Rotamer outliers (%)	2.01	3.05	2.70
Twin operator and fraction		-H,-K,L/0.360	

[a] Protein monomers/DNA duplexes
[b] Number of isotropic / anisotropic B-factors
[c] Number of atoms not belonging to protein, to DNA and to bound MQ.

Supplementary Table 8. Refinement statistics of R282W-related structures

Structure	**R282W-DNA-MQ**	**R282W-DNA (I)**	**R282W-DNA (II)**
PDB code	7B4E	7B4F	7B4G
Resolution range (Å)	34.1-1.57	18.4-1.78	34.3-1.86
No. of reflections (*I*> 0)	30,182	21,071	18,811
No. of reflections in test set	2,009	2,108	1,897
No. of molecules in the a.u.[a]	1/0.5	1/0.5	1/0.5
R_{work} (%) / R_{free} (%)	16.6/20.1	15.5/19.5	14.7/20.5
No. of TLS groups	7	9	5
No. iso/aniso B-factors[b]	2323/1	2149/0	2290/0
No. of protein / DNA atoms	1704/266	1634/206	1707/226
No. of solvent atoms[c]	314	309	357
No. of MQ conjugates (atoms)	4 (40)	-	-
<*B* factors> (Å^2)			
- Overall	21.8	24.6	21.7
- Protein / DNA / Solvent	18.1/34.0/28.7	23.4/24.3/31.1	20.5/21.1/27.6
- MQ conjugates	41.3	-	-
Root mean square deviations:			
- Bond length (Å) / Angle (°)	0.006/1.05	0.004/0.72	0.007/0.95
Ramachandran Plot:			
- Favored (%)	100	99.5	99.0
- Outliers (%)	0.0	0.0	0.0
Rotamer outliers (%)	1.55	1.09	3.63

[a] Protein monomers/DNA duplexes

[b] Number of isotropic / anisotropic B-factors

[c] Number of atoms not belonging to protein, to DNA and to bound MQ.

Supplementary Table 9. Refinement statistics of wt-DNA-MQ structures				
Structure	**wt-DNA-MQ (I)**	**wt-DNA-MQ (II)**	**wt-DNA-MQ (III)**	**wt-DNA-MQ (P1)**
PDB code	6ZNC	7B4N	7B4H	7B46
Resolution range (Å)	46.6-1.64	47.0-1.32	47.1-1.39	47.7-2.00
No. of reflections (*I*> 0)	27,440	53,673	46,784	58,628
No. of reflections in test set	1,933	3,756	2,352	1,998
No. of molecules in the a.u.[a]	1/0.5	1/0.5	1/0.5	4/2
R_{work} (%) / R_{free} (%)	16.0/19.0	14.0/16.6	16.9/20.0	17.9/20.3
No. of TLS groups	8	0	0	40
No. iso/aniso B-factors[b]	2096/1	333/1967	785/1438	7337/0
No. of protein / DNA atoms	1605/273	1669/291	1656/217	6051/900
No. of solvent atoms[c]	179	310	320	306
No. of MQ conjugates (atoms)	4 (40)	3 (30)	3 (30)	8 (80)
<*B* factors> ($Å^2$)				
- Overall	27.5	23.1	24.8	47.7
- Protein / DNA / Solvent	25.4/35.2/32.5	19.8/30.8/32.3	22.3/32.7/30.0	44.6/69.5/41.9
- MQ conjugates	40.0	37.6	52.7	58.6
Root mean square deviations:				
- Bond length (Å) / Angle (°)	0.011/1.23	0.008/1.057	0.004/0.88	0.007/0.869
Ramachandran Plot:				
- Favored (%)	100	99.46	98.94	99.1
- Outliers (%)	0.0	0.0	0.0	0.1
Rotamer outliers (%)	2.89	2.10	1.57	3..49

[a] Protein monomers/DNA duplexes
[b] Number of isotropic / anisotropic B-factors
[c] Number of solvent atoms not belonging to protein, to DNA and to bound MQ.

Supplementary Table 10. Schematic presentations of the DNA response elements of the p53-DNA complexes

Structure	Space group	A/T pairs	DNA duplex
R273H-DNA-MQ	*C*2	WC	T<br>\\<br>$-C^{19}-C^{20}\ G^{1}-G^{2}-G^{3}-C^{4}-A^{5}-T^{6}-G^{7}-C^{8}-C^{9}-C^{10}-G^{11}-G^{12}-G^{13}-C^{14}-A^{15}-T^{16}-G^{17}-C^{18}-C^{19}-C^{20}\ G^{1}-G^{2}-$<br>$-G^{2}-G^{1}\ C^{20}-C^{19}-C^{18}-G^{17}-T^{16}-A^{15}-C^{14}-G^{13}-G^{12}-G^{11}-C^{10}-C^{9}-C^{8}-G^{7}-T^{6}-A^{5}-C^{4}-G^{3}-G^{2}-G^{1}\ C^{20}-C^{19}-$<br>\\<br>T
R273H-DNA	*C*2	WC	T<br>\\<br>$-C^{19}-C^{20}\ G^{1}-G^{2}-G^{3}-C^{4}-A^{5}-T^{6}-G^{7}-C^{8}-C^{9}-C^{10}-G^{11}-G^{12}-G^{13}-C^{14}-A^{15}-T^{16}-G^{17}-C^{18}-C^{19}-C^{20}\ G^{1}-G^{2}-$<br>$-G^{2}-G^{1}\ C^{20}-C^{19}-C^{18}-G^{17}-T^{16}-A^{15}-C^{14}-G^{13}-G^{12}-G^{11}-C^{10}-C^{9}-C^{8}-G^{7}-T^{6}-A^{5}-C^{4}-G^{3}-G^{2}-G^{1}\ C^{20}-C^{19}-$<br>\\<br>T
R282W-DNA-MQ	*C*2	HG+WC	$C^{11}-G^{12}$ $C^{11}-G^{12}$<br>/ /<br>$C^{1}-G^{2}-G^{3}-G^{4}-C^{5}-A^{6}-T^{7}-G^{8}-C^{9}-C^{10}\ C^{1}-G^{2}-G^{3}-G^{4}-C^{5}-A^{6}-T^{7}-G^{8}-C^{9}-C^{10}\ C^{1}-G^{2}-$<br>$-G^{2}-C^{1}\ C^{10}-C^{9}-G^{8}-T^{7}-A^{6}-C^{5}-G^{4}-G^{3}-G^{2}-C^{1}\ C^{10}-C^{9}-G^{8}-T^{7}-A^{6}-C^{5}-G^{4}-G^{3}-G^{2}-C^{1}$<br>/ /<br>$G^{12}-C^{11}$ $G^{12}-C^{11}$
R282W-DNA (I)	*C*2	HG	$C^{11}-G^{12}$ $C^{11}-G^{12}$<br>/ /<br>$C^{1}-G^{2}-G^{3}-G^{4}-C^{5}-A^{6}-T^{7}-G^{8}-C^{9}-C^{10}\ C^{1}-G^{2}-G^{3}-G^{4}-C^{5}-A^{6}-T^{7}-G^{8}-C^{9}-C^{10}\ C^{1}-G^{2}-$<br>$-G^{2}-C^{1}\ C^{10}-C^{9}-G^{8}-T^{7}-A^{6}-C^{5}-G^{4}-G^{3}-G^{2}-C^{1}\ C^{10}-C^{9}-G^{8}-T^{7}-A^{6}-C^{5}-G^{4}-G^{3}-G^{2}-C^{1}$<br>/ /<br>$G^{12}-C^{11}$ $G^{12}-C^{11}$
R282W-DNA (II)	*C*2	HG	$C^{11}-G^{12}$ $C^{11}-G^{12}$<br>/ /<br>$C^{1}-G^{2}-G^{3}-G^{4}-C^{5}-A^{6}-T^{7}-G^{8}-C^{9}-C^{10}\ C^{1}-G^{2}-G^{3}-G^{4}-C^{5}-A^{6}-T^{7}-G^{8}-C^{9}-C^{10}\ C^{1}-G^{2}-$<br>$-G^{2}-C^{1}\ C^{10}-C^{9}-G^{8}-T^{7}-A^{6}-C^{5}-G^{4}-G^{3}-G^{2}-C^{1}\ C^{10}-C^{9}-G^{8}-T^{7}-A^{6}-C^{5}-G^{4}-G^{3}-G^{2}-C^{1}$<br>/ /<br>$G^{12}-C^{11}$ $G^{12}-C^{11}$

R273C/S240R-DNA-MQ	*C*2	WC	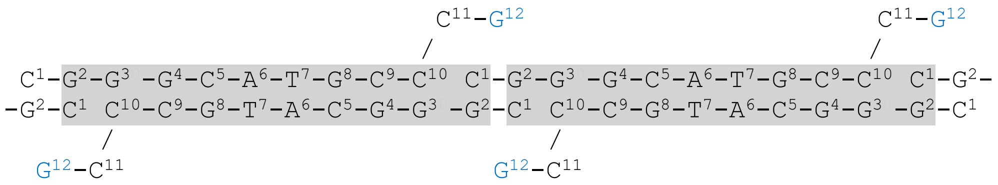
R273C/S240R-DNA[a]	*C*2	WC	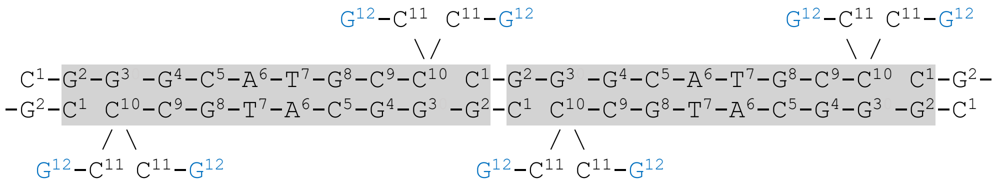
wt-DNA-MQ (I)	*C*2	HG	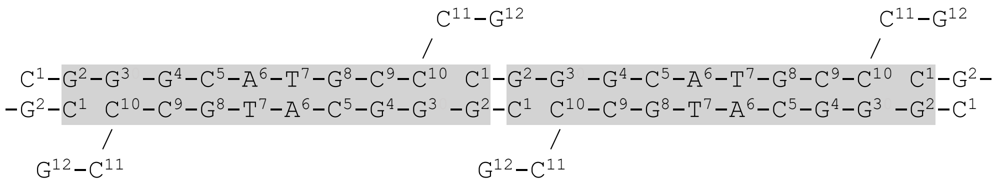
wt-DNA-MQ (II)	*C*2	HG	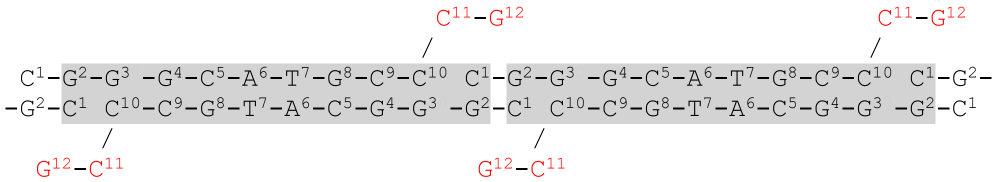
wt-DNA-MQ (III)	*C*2	HG	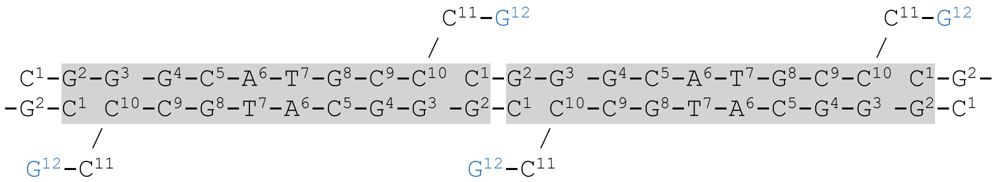
wt-DNA[b]	*C*2	HG	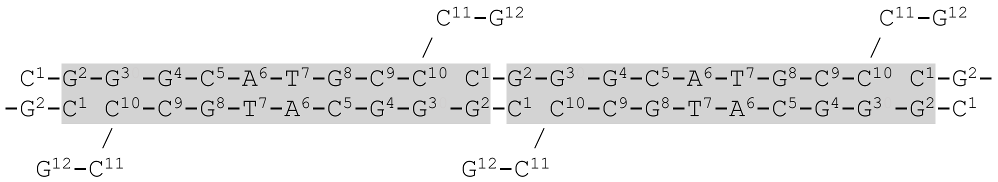

wt-DNA-MQ (P1)	*P*1	WC	C^{1}-G^{2}-G^{3}-G^{4}-C^{5}-A^{6}-T^{7}-G^{8}-C^{9}-C^{10}-C^{11}-G^{12} C^{1}-G^{2}-G^{3}-G^{4}-C^{5}-A^{6}-T^{7}-G^{8}-C^{9}-C^{10}-C^{11}-G^{12}<br>G^{12}-C^{11}-C^{10}-C^{9}-G^{8}-T^{7}-A^{6}-C^{5}-C^{4}-C^{3}-G^{2}-C^{1} G^{12}-C^{11}-C^{10}-C^{9}-G^{8}-T^{7}-A^{6}-C^{5}-G^{4}-G^{3}-G^{2}-C^{1}
wt-DNA (P1)[c]	*P*1	WC	C^{1}-G^{2}-G^{3}-G^{4}-C^{5}-A^{6}-T^{7}-G^{8}-C^{9}-C^{10}-C^{11}-G^{12} C^{1}-G^{2}-G^{3}-G^{4}-C^{5}-A^{6}-T^{7}-G^{8}-C^{9}-C^{10}-C^{11}-G^{12}<br>G^{12}-C^{11}-C^{10}-C^{9}-G^{8}-T^{7}-A^{6}-C^{5}-C^{4}-C^{3}-G^{2}-C^{1} G^{12}-C^{11}-C^{10}-C^{9}-G^{8}-T^{7}-A^{6}-C^{5}-G^{4}-G^{3}-G^{2}-C^{1}

The 20 base-pair long DNA response elements are made of 21-mer oligomers for R273H-DNA complexes, and of 12-mer oligomers for all other complexes (see Methods). The two decameric half-sites are highlighted by a grey background. In the half-sites made by the 12-mer oligomers (space group C2) two nucleotide bases at each 3'-end (C11-G12) are extra-helical and the continuous 20-bp duplex is made by base-stacking interactions between C10 of one duplex and C1 of an adjacent duplex (related by translational symmetry along the crystal c axis). Unresolved bases are in blue color. Bases in red color for R273H-DNA-MQ indicate two alternative backbone conformations between C9 and C10 and the equivalent C19 and C20 bases induced by MQ binding (see text for details). Bases in orange for wt-DNA-MQ (II) indicate three alternative backbone conformations. WC refers to Watson-Crick base pairs. HG refers to Hoogsteen base pairs.

[a] PDB ID 4IBV (Eldar *et al.*, 2013)[1]
[b] PDB ID 3IGL (Kitayner *et al.*, 2010)[2]
[c] PDB ID 2AC0 (Kitayner *et al.*, 2006)[3]

Supplementary Table 11. Unresolved residues in L1 loop (113-124)			
Structure	Space group[a]	Missing monomer residues[b]	Partially defined residues[c]
R273H-DNA-MQ	*C*2 (2)	**A** (116-118)<br>**B** (116-118)	**A** (115)$^{0.79}$ **A** (119)$^{0.61}$ **A** (120-121)$^{0.39}$ **A** (122)$^{0.67}$<br>**B** (115)$^{0.81}$ **B** (119)$^{0.60}$ **B** (120-121)$^{0.39}$ **B** (122)$^{0.69}$
R273H-DNA	*C*2 (2)	None	**A** (117-118)$^{0.51}$<br>**B** (117-118)$^{0.52}$
R282W-DNA-MQ	*C*2 (1)	**A** (114-121)	
R282W-DNA (I)	*C*2 (1)	None	
R282W-DNA (II)	*C*2 (1)	None	
R273C/S240R-DNA-MQ	C2 (1)	None	
R273C/S240R-DNA[d]	C2 (1)	None	**A** (115)$^{0.91}$
wt-DNA-MQ (I)	*C*2 (1)	None	**A** (114-121)$^{0.89}$
wt-DNA-MQ (II)	*C*2 (1)	**A** (117-118) **A** (121)	**A** (115)$^{0.65}$ **A** (116)$^{0.52}$ **A** (119)$^{0.66}$ **A** (120)$^{0.89}$
wt-DNA-MQ (III)	*C*2 (1)	**A** (117-119)	
wt-DNA[e]	*C*2 (1)	None	
wt-DNA-MQ (P1)	*P*1 (4)	**B** (116-117)<br>**D** (117-119)	**B** (120)$^{0.89}$ **B** (121)$^{0.80}$
wt-DNA (P1)[f]	*P*1 (4)	None	

[a] The number of protein monomers in the asymmetric units are in parentheses.
[b] The upper case letter (in bold) refer to the protein monomer. The residues' numbers are in parentheses.
[c] The refined occupancy values of the residues' main-chain atoms are shown in superscript.
[d] PDB ID 4IBV [1]
[e] PDB ID 3IGL [2]
[f] PDB ID 2AC0 [3]

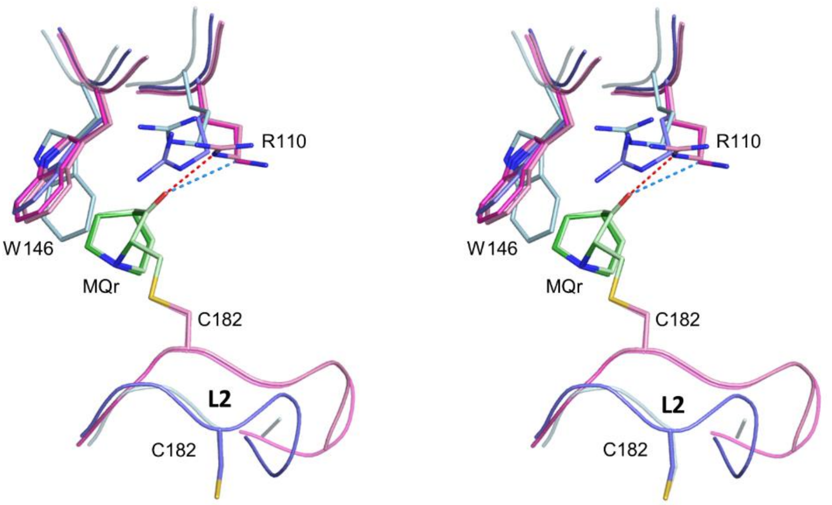

Supplementary Fig. 1. Divergent stereo view of superposed two conformations of L2 loops at C182 residues in the presence and absence of bound MQ.

An outward L2 conformation (toward the solvent) is observed at C182 residues bound to MQ, represented by monomers B of R273H-MQ (I) and R273C-MQ (I) structures (shown in pink and magenta, respectively). An inward L2 conformation (toward the protein) is observed at unmodified C182 residues, represented by monomers A of the same structures (shown in blue and light blue, respectively). Also shown W146 and R110 residues from neighboring monomers that stabilize the MQ-C182 conjugates at the outward L2 conformation by stacking and electrostatic interactions, respectively (see also **Fig. 1**). The protein backbones are in cartoon representation and the amino-acid side chains in stick representation. Color codes for C atoms are as for the corresponding cartoons and green for MQ. N, O and S atoms are in blue, red and yellow, respectively. The later color codes for atoms are common to all figures. Hydrogen bonds and CH···O interactions indicated by red and blue dotted lines, respectively.

a

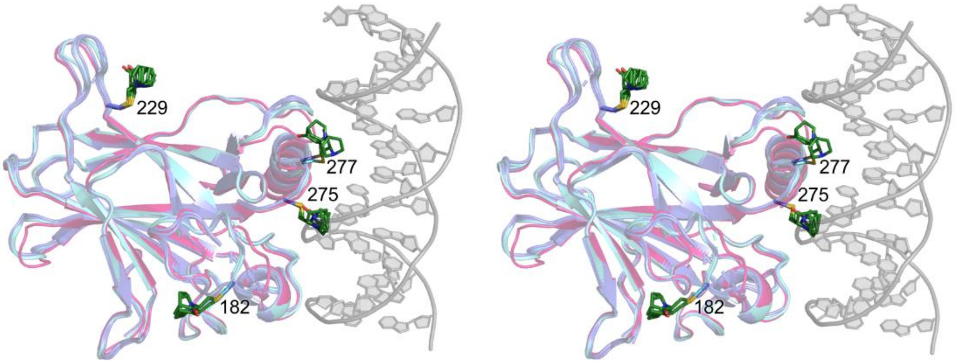

b

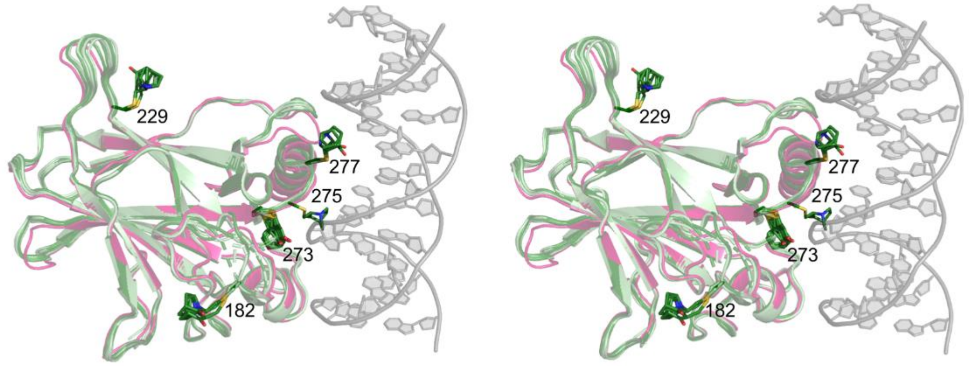

Supplementary Fig. 2. Comparison of MQ-modified p53 monomers from R273H-MQ and R273C-MQ structures with the structure of wild-type p53DBD bound to DNA.

(a) A stereo view of superposition of all modified monomers (see **Table 1**) from R273H-MQ (I) and R273H-MQ (II) structures (shown in light blue shades), with wt p53DBD bound to DNA (PDB ID 5MCV[4] (shown in magenta and grey, respectively). **(b)** Superposition of all modified monomers from R273C-MQ (I) and R273C-MQ (II) structures (shown in light green shades), with wt-DNA structure (PDB ID 5MCV, shown in magenta and grey, respectively). The comparisons show that MQ conjugates at C182, C229, C277 and C273 in the two DNA-contact mutants are compatible with DNA binding whereas MQ-C275 is not, as it "penetrates" into the space occupied by the DNA (see main text for details). Protein monomers and DNA helix are in cartoon representations. MQ-Cys conjugates are in stick representation and labeled.

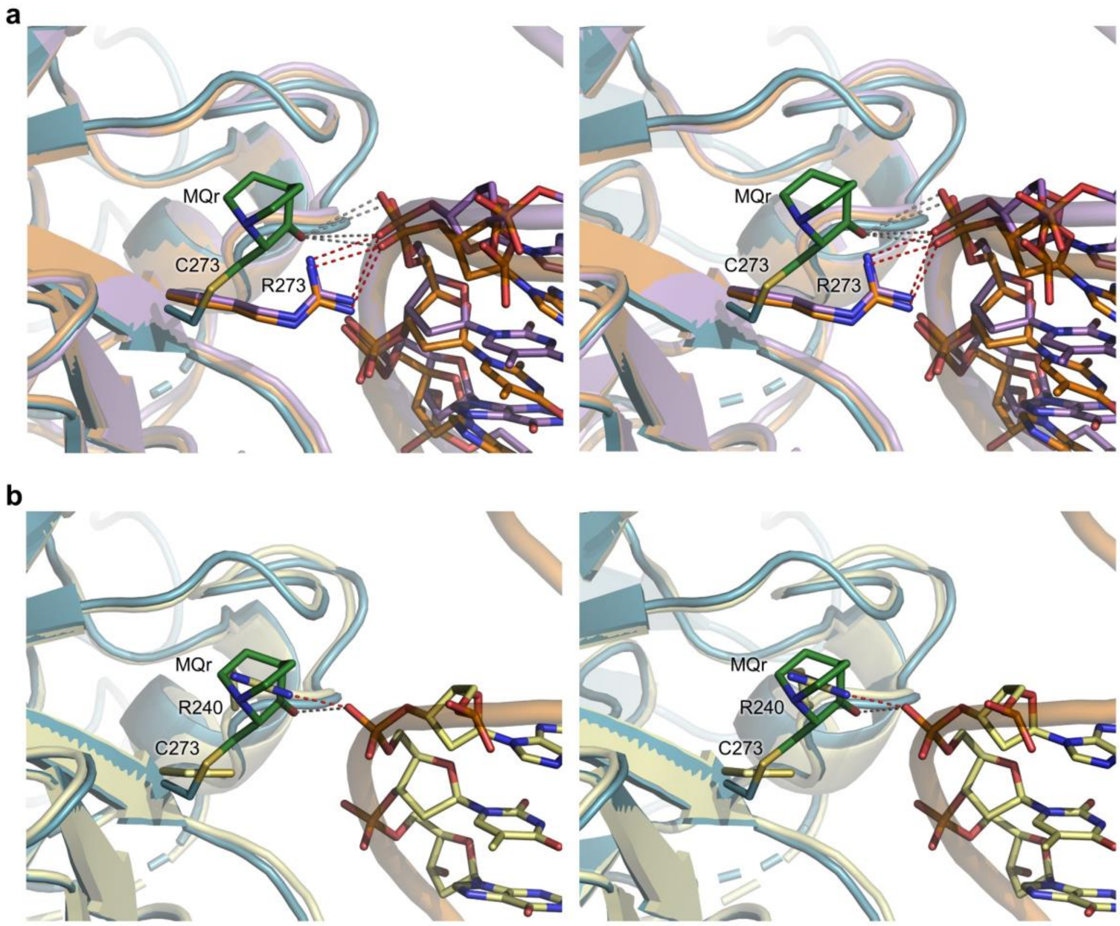

Supplementary Fig. 3. Compatibility of MQ-C273 conjugates with DNA binding.

(a) A stereo view of monomer D from R273C-MQ (I) structure (light blue), incorporating an MQ-C273 conjugate (of high occupancy level), compared by superposition with monomers of two types of wt p53-DNA complexes (PDB ID 2AC0[3], in orange, and PDB ID 5MCW[4], in violet). Both wt structures show all Watson-Crick base pairs which are characteristic of p53-DNA complexes incorporating the DNA-contact C273 mutation (Eldar *et al.*, 2013)[1]. In this model, the distances between MQ-C273 (via the carbonyl oxygen of MQ) and the phosphate-backbone oxygens range from 3.5 to 3.8 Å (shown as grey dotted lines). Also shown, R273 side chains from the two wt p53-DNA structures forming hydrogen bonds with the phosphate oxygens of the corresponding DNA backbones (shown as red dotted lines). Protein and DNA backbones are in cartoon representation. R273 side chains, MQ-C273 conjugate and DNA nucleosides are in stick representation.

(b) A stereo view of the same monomer of R273C-MQ (I) shown in (a), compared by superposition with a monomer from R273C/S240R-DNA-MQ structure (in yellow). It shows that MQ-C273 is positioned in the space occupied by the second-site suppressor mutation R240 of the complex forming an alternative hydrogen bond with the phosphate oxygen of the DNA backbone (shown as red dotted line). This explains why C273 is not modified by MQ in the double-mutant complex. In this model, the distance between the carbonyl oxygen of MQ bound to C273 and the same phosphate oxygen is 2.8 Å (shown as a grey dotted line). Structure representations and color codes as in (a).

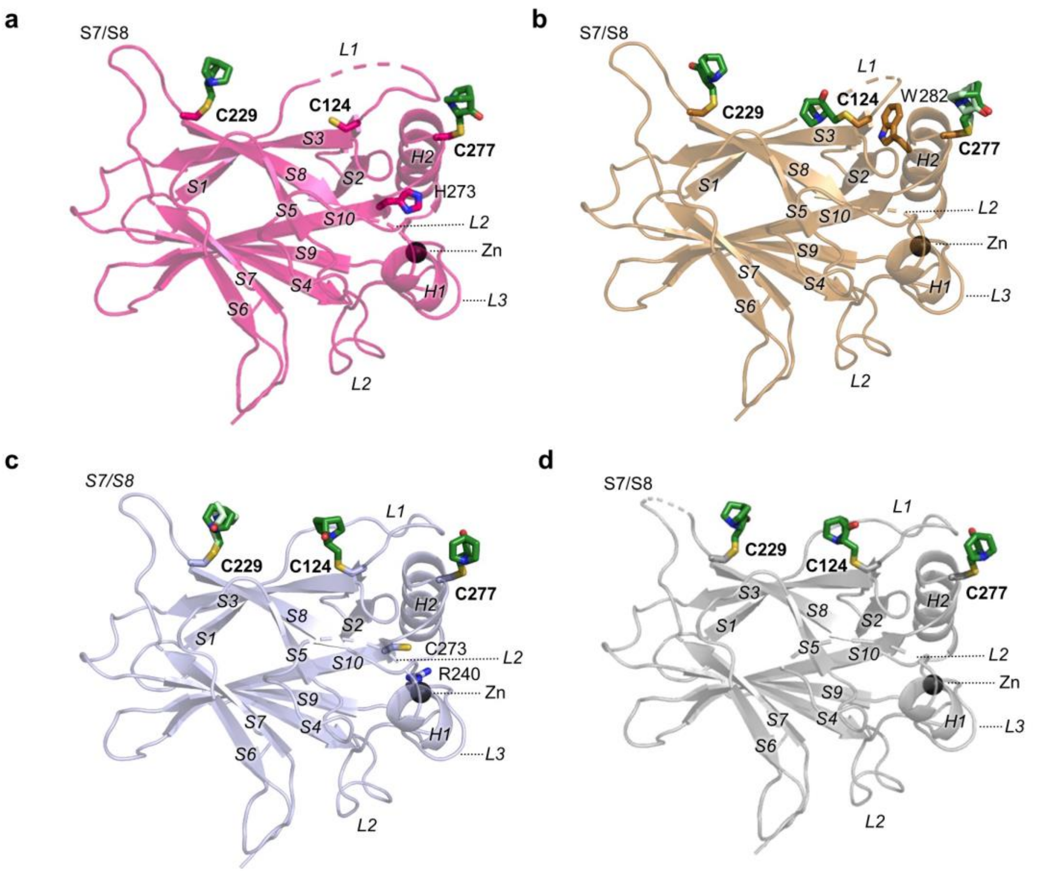

Supplementary Fig. 4. Overall views of MQ-Cys conjugates within their core domains observed in p53-DNA-MQ structures.

The presented p53 core domains bound to MQ are from the following tetrameric structures shown in **Fig. 6**. **(a)** R273H-DNA-MQ. **(b)** R282W-DNA-MQ. **(c)** R273C/S240R-DNA-MQ. **(d)** wt-DNA-MQ (I). The overall views show the positions of MQ-C124, MQ-C229 and MQ-C277 conjugates relative to other regions of the core domain. Two bound MQ enantiomers (MQr in light green and MQs in dark green) are shown by MQ-C277 in (b) and MQ-C229 in (c). The protein is represented as a cartoon. Unresolved regions of L1 and L2 loops are shown as dashed lines. MQ-Cys conjugates and mutated residues are shown as sticks. Zn ions are shown as black spheres.

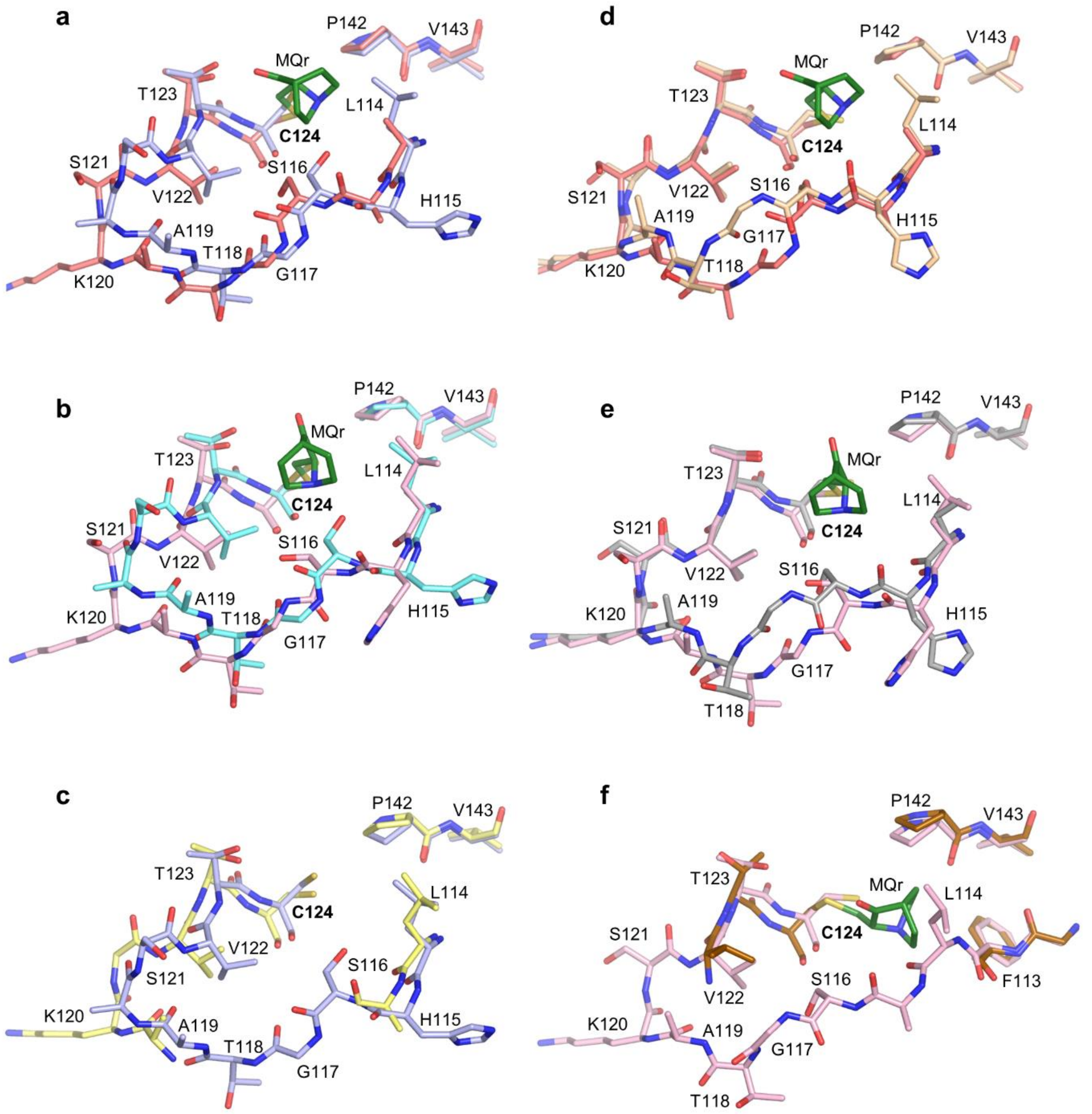

Supplementary Fig. 5. Close up views of the accessible space around C124 residues in free and DNA-bound p53 core domains.

Space comparisons are shown in two groups: (**a-c**) MQ-C124 conjugates in p53-DNA-MQ structures versus unmodified C124 in structures of the free modified mutants (R273H-MQ or R273C-MQ), and (**d-f**) MQ-C124 conjugates in p53-DNA-MQ structures versus C124 in the corresponding unmodified p53-DNA complexes. The superposition views (based on Cα atoms of the core domains), compare the space around C124 between p53 monomers from the following pairs of structures.

(a) wt-DNA-MQ (I) (salmon) and R273H-MQ (I) (monomer A in violet).

(b) R273C/S240R-DNA-MQ (pink) and R273C-MQ (I) (monomer A in cyan).

Supplementary Fig. 5 (cont.)

(c) R273H-DNA-MQ (monomer A in yellow) and R273H-MQ (I) (monomer A in violet). MQ-C124 is likely present in R273H-DNA-MQ in view of an un-modelled electron density bulk close to C124 (see **Table 1**). Residues 116-118 of R273H-DNA-MQ are not resolved in the crystal structure.

(d) wt-DNA-MQ (I) (salmon) and wt-DNA (PDB ID 3IGL[2], in light brown).

(e) R273C/S240R-DNA-MQ (pink) and R273C/S240R-DNA (PDB ID 4IBV[1] in grey).

(f) R282W-DNA-MQ (copper) and R282W-DNA (I) (in pink). Residues 114-121 of R282W-DNA-MQ are not resolved in the crystal structure.

The first group of structures shows that the space around C124 is significantly larger in the MQ-modified p53-DNA complexes than that of the free MQ-modified mutants, resulting from a local conformational change of the L1 loop (residues 116-124) in the complexes toward the DNA. This change is an intrinsic feature of most p53-DNA complexes due to the interaction of K120 side chain with the DNA (shown in **Fig. 9**). By contrast, K120 side chains are largely disordered in all monomers of the DNA-free structures. The second group of structures shows that the space around C124 residues is similar among the MQ-modified and unmodified p53-DNA complexes, except for that of R282W-DNA-MQ **(f).** In the later structure, MQ-C124 conjugate displays a large change in orientation and position towards F113 (shown also in **Fig. 7a**), associated with a major disorder of L1 loop (see Supplementary **Table 11** and main text for details).

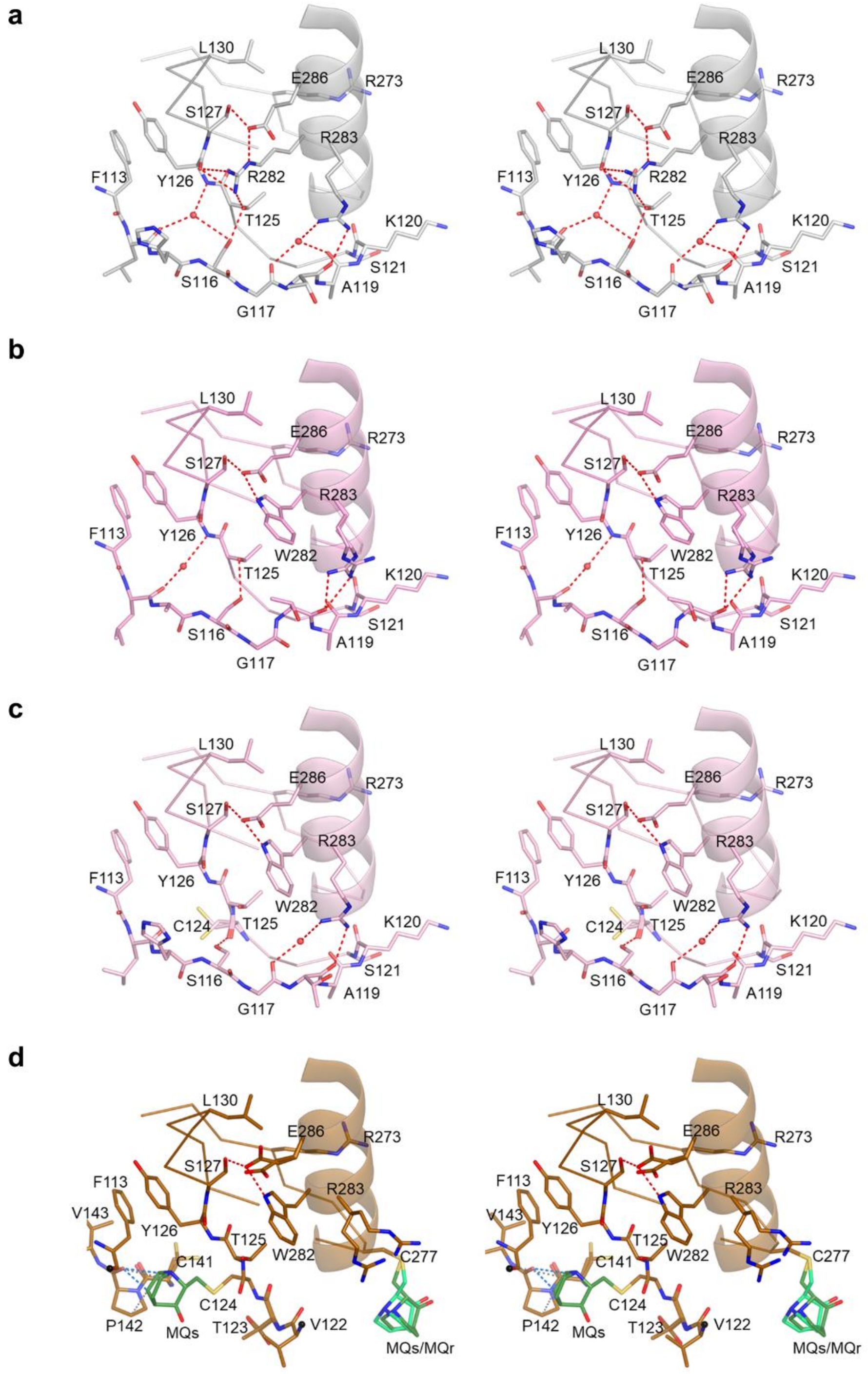

Supplementary Fig. 6. Stereo views of local conformational changes in wt p53 bound to DNA caused by replacing R282 by W282 and by MQ binding to R282W-DNA.

(a) wt-DNA (PDB ID 3IGL[2]). R282 is located on the H2 helix and stabilizes the surrounding regions by anchoring a network of hydrogen bonds.

(b, c) R282W-DNA (I, II). The replacement of R282 by W282 results in diminished stabilizing contacts and two alternative conformations of L1 loop (residues 113-124). Whereas the free R282W protein is unstable, its complex with DNA is stable (see main text for details).

Supplementary Fig. 6 (cont.)

(d) R282W-DNA-MQ. Two MQ-Cys conjugates are located at the regions affected by W282. MQ-C124 is at the C-terminus of L1, and MQ-C277 (showing two alternative enantiomers) is near the N-terminus of the H2 helix. MQ-C124 forms CH···O and VdW interactions with residue P142 shown here, as well as other interactions shown in **Fig. 7a**. MQ-C277 forms stabilizing interactions with the DNA shown in **Fig. 9b**. This modification leads to largely disordered L1 loop at residues 114-121 (see main text for details). Two alternative conformations are shown by amino acids R283, E286 and C141 (**b, d**).

L1 loop (residues 113-124) and adjacent regions are in stick representation. H1 helix is in cartoon with specific residues in stick representation. Water molecules are shown as red spheres. Hydrogen bonds and CH···O interactions are shown as red and blue dotted lines, respectively.

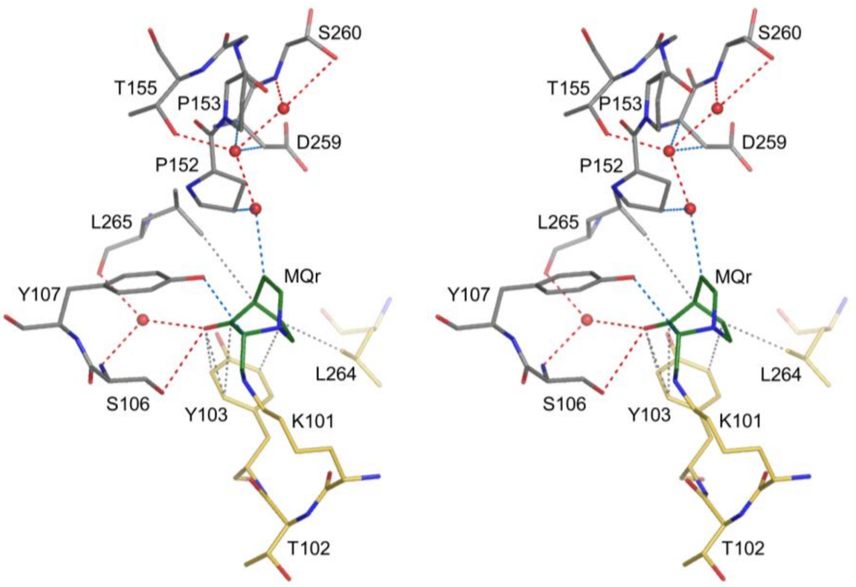

Supplementary Fig. 7. A stereo view of MQ bound to K101 in wt-DNA-MQ (P1) structure.

The MQ-Lys conjugate, observed in monomer A, is stabilized extensively by intramolecular interactions and by intermolecular interactions from a symmetry-related monomer C. These interactions include direct and water-mediated hydrogen bonds and CH···O interactions, as well as VdW interactions.

The single MQ-Lys adduct observed in the current study, was trapped in the crystal of wt-DNA-MQ (P1) by stabilizing interactions between two neighboring p53 monomers from two p53-DNA tetramers (see text for details).

The structure is in stick representation, and the color codes for C atoms are yellow and grey for monomer A and symmetry-related monomer C, respectively. Other color codes are as in Supplementary **Fig. 1**. Water molecules are shown as red spheres. Hydrogen bonds, CH···O and VdW interactions shown in red, blue and grey dotted lines, respectively.

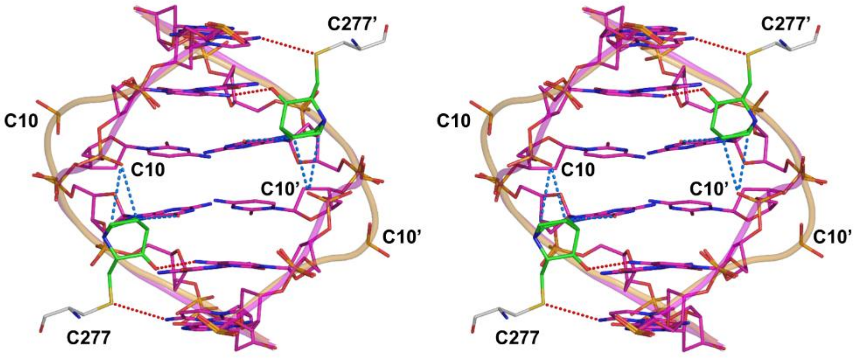

Supplementary Fig. 8. **A close-up stereo view of the interactions of two symmetry-related MQ-C277 conjugates with the DNA helix in R273H-DNA-MQ structure.**

The view highlights the change in the positions of two symmetry-related phosphate groups of C10 and C10' nucleotides towards the central major groove relative to that of the unmodified complex. This conformational change in DNA leads to CH···O interactions with the two symmetry-related MQ-C277 conjugates. Also shown are MQ-mediated hydrogen bonds with the DNA bases (see also **Figs. 6, 9a**, and the main text for details). Bound MQ conjugates and DNA are in stick representation. Also shown in cartoon, the DNA backbone (based on the phosphate coordinates) of the modified complex (magenta) superposed on that of the unmodified complex (light brown). Hydrogen bonds and CH···O interactions indicated by red and blue dotted lines, respectively.

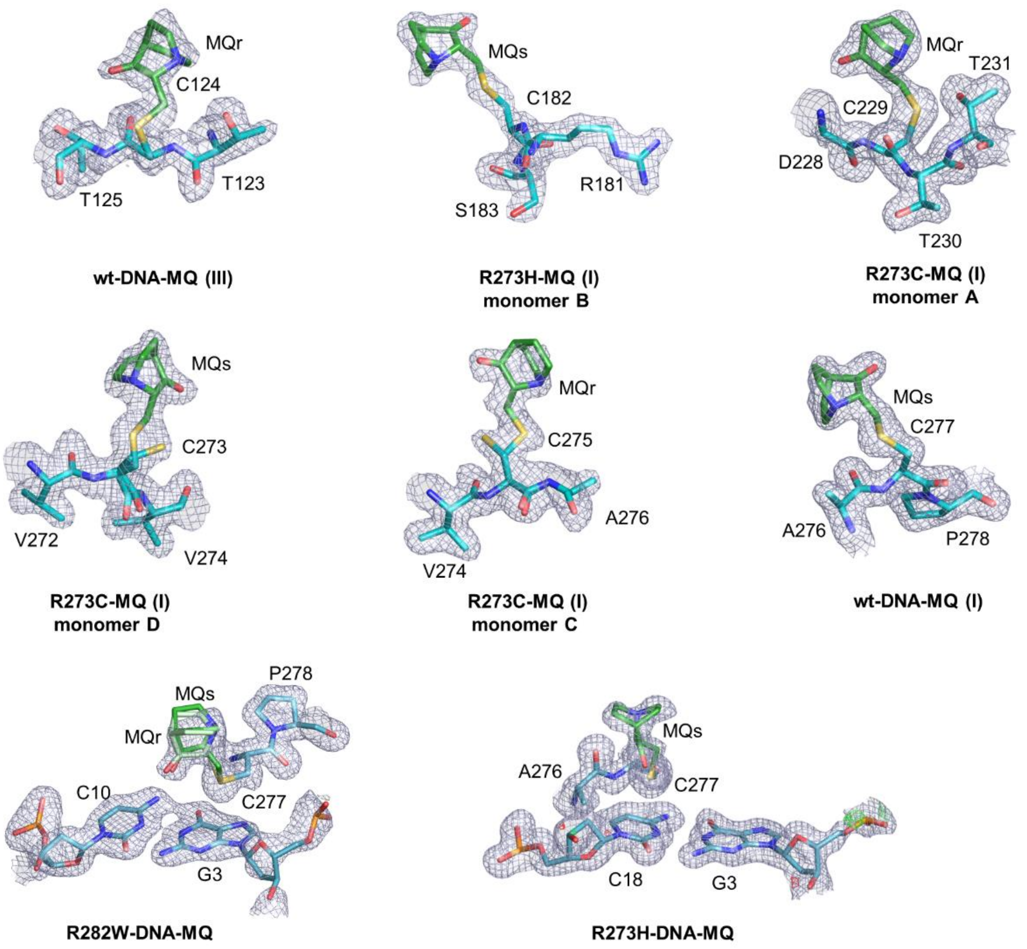

Supplementary Fig. 9. MQ-Cys conjugates and nearby amino acids and nucleotides shown within their electron density maps (2mF$_o$-DF$_c$ contoured at 1.0 σ level).

Supplementary Discussion

Does the structure-based study assess the potential rescue of specific cysteines based on the assays by Zhang *et al*. (2018)

The role of specific Cys residues in stabilizing and reactivating mutant p53 has been studied by biochemical and cell-based assays, respectively. Zhang *et a*l.[5] conducted thermal stability measurements of the core domains of wt p53, R273H and R175H proteins where the wt cysteines at positions: 124, 277, 182, 229 and 124/277, were replaced by alanines. The data showed that the replacement of C277 by A277 led to the largest reduction in MQ-mediated thermal stabilization, compared to that of the other Cys to Ala replacements, thus indicating that MQ binding to C277 contributes to thermo-stabilization of the p53 core domain.

The current crystal structures of the isolated core domains of R273H and R273C mutants show that MQ-C277 conjugates form stabilizing contacts with the proteins (**Fig. 4**) and hence likely contribute to increased p53 stability. By contrast, well-defined MQ-C124 conjugates were not resolved in the structures of the two mutants, other than electron density traces (**Table 1**), suggesting that their contribution to p53 stability in the DNA-free state may not be significant. However, determining the role of MQ-C124 based on thermal stability is complicated as the replacement of C124 by A124 led to different effects in different proteins. The potential role of MQ-C182 on p53 stabilization cannot be confirmed by the structural data because stabilizing contacts are made only with neighboring monomers in the crystal (**Table 2**). MQ-C229 conjugates in the same structures show high occupancy levels and mutual stabilization between MQ and p53 (**Tables 1, 2**), and hence likely contribute to local stabilization of the core domains of these mutants, whereas the data of Zhang *et al.*, showed only a slight but consistent reduction in thermal stability as a result of replacing C229 by A229 in all three proteins. However, effects caused merely by replacing cysteines by alanines could affect the thermal stability data, as exemplified by replacing C124 by A124.

To investigate the role of cysteines on mutant p53 reactivation by APR-246/MQ in tumor cells, Zhang *et al*. used vectors encoding the p53 mutants: R175H, R175H/C124A, R175H/C277A or R175H/C124A/C277A. Based on these data, they proposed that both C124 and C277 were required for MQ-mediated reactivation of R175H in tumor cells[5]. Our structural data of MQ-modified p53-DNA complexes suggest that three MQ-modified residues, C124, C229 and C277, contribute to mutant p53 reactivation. The role of MQ-C124 and MQ-C277 is in accordance with the cell-based assay, whereas the potential role of MQ-C229 was not tested yet. The role of MQ-C124 in transcriptional reactivation of R175H was also proposed by Wassman *et al*.[6]

Specific p53 cancer mutants that may be more amenable to rescue by MQ than others.

On the basis of the available structural data, including the current MQ-modified structures, the previously published structures of wt and mutant p53, and the published data on mutant p53 reactivation, we propose the potential effects of MQ on other p53 cancer mutants.

The current MQ-modified structures demonstrate that DNA binding of both DNA-contact and structural mutants is supported by a general mechanism mediated by MQ bound to three cysteines (C124, C229 and C277). In addition to R273H bound to both DNA and MQ

studied here, it is likely that DNA binding activity of other DNA-contact mutants, including R248Q, R280K and R273C, is restored by the same mechanism where the loss of hydrogen bonds with DNA is compensated for by new MQ-mediated p53-DNA and protein-protein interactions. DNA-binding of these mutants were also shown previously to be restored by PRIMA-1[7].

Whereas DNA-contact mutants are mostly thermodynamically stable, the structural mutants display various levels of stability and folding ranging from weakly destabilized and locally distorted mutants (e.g. G245S and R249S), to globally denatured mutants (e.g. R175H and R282W)[8]. The current structural data show that the structural mutant R282W displays the common MQ-mediated mechanism, so it is likely that a similar mechanism leads to R175H reactivation. Both mutants were shown to be rescued by APR-246 or PRIMA-1[5-7].

Zn ions play a major role in the stability of the p53 core domains and their complexes with DNA[3,9]. In the structures of p53-DNA tetramers, two central water molecules in each dimer provide a central anchor for an internal hydrogen-bonding network that links the two Zn ions, each coordinated to C176, C238, C242 and H179, thereby supporting the H1 helix and L2 and L3 loops, involved in maintaining the protein-DNA and the protein-protein interfaces within and between p53 dimers[2,3]. Hence, mutations that directly or indirectly impair the Zn coordination would weaken the stability of the tetrameric p53-DNA complex as shown by certain p53 mutants.

The crystal structures of the cancer mutants G245S and R249S display local structural distortions near the sites of the specific mutations[10,11,12] where the corresponding mutations, S245 or S249, located at the L3 loop, differently affect the tetrahedral Zn coordination. In G245S, the hydroxyl side chain of S245 interacts with the thiol group of C238, leading to a local distortion of L3 at residues M243-G244. In R249S, the replacement of R249 by S249 results in the loss of a salt bridge and hydrogen bonds between L2 and L3 loops, as well as distorting the Zn coordination by the formation of two alternative conformations of C238[11]. DNA binding activity of these and of other mutants was shown to be restored by the presence of second-site mutations presenting alternative stabilizing interactions[1,10,11]. Hence, such mutants might be rescued by the combined mechanism shown by the current p53-DNA-MQ structures. In the case of G245S, it was also shown that PRIMA-1 led to significant reactivation of PUMA and p21 transcriptional reporters[6]. However, unlike local structural distortions in the Zn regions, direct mutations at the Zn coordination site could lead to global denaturation of p53. Examples of such mutants are C242S which is largely unfolded[8], not studied yet for APR-246/MQ-mediated reactivation, and C176F being the only exception out of 14 mutants, not showing DNA-binding activity after PRIMA-1 treatment[7].

The ultimate assessment of MQ-mediated functional rescue of p53 oncogenic mutants will be achieved by structural data combined with quantitative functional assays using a large range of p53 response elements.

Supplementary References

1. Eldar, A., Rozenberg, H., Diskin-Posner, Y., Rohs, R. & Shakked, Z. Structural studies of p53 inactivation by DNA-contact mutations and its rescue by suppressor mutations via alternative protein-DNA interactions. *Nucleic Acids Res* 41, 8748-7859 https://doi.org/10.1093/nar/gkt630 (2013).

2. Kitayner, M. et al. Diversity in DNA recognition by p53 revealed by crystal structures with Hoogsteen base pairs. *Nat Struct Mol Biol* 17, 423-429 https://doi.org/10.1038/nsmb.1800 (2010).

3. Kitayner, M. et al. Structural basis of DNA recognition by p53 tetramers. *Mol. Cell* 22, 741-753 https://doi.org/10.1016/j.molcel.2006.05.015 (2006).

4. Golovenko, D., *et al.* New Insights into the Role of DNA Shape on Its Recognition by p53 Proteins. *Structure* **26**, 1237-1250 e1236. https://doi.org/10.1016/j.str.2018.06.006 (2018).

5. Zhang, Q., Bykov, V.J.N., Wiman, K.G. & Zawacka-Pankau, J. APR-246 reactivates mutant p53 by targeting cysteines 124 and 277. *Cell Death Dis* 9, 439 https://doi.org/10.1038/s41419-018-0463-7 (2018).

6. Wassman, C.D. et al. Computational identification of a transiently open L1/S3 pocket for reactivation of mutant p53. *Nat Commun* 4, 1407 https://doi.org/10.1038/ncomms2361 (2013).

7. Bykov, V.J. et al. Restoration of the tumor suppressor function to mutant p53 by a low-molecular-weight compound. *Nat Med* 8, 282-288 https://doi.org/10.1038/nm0302-282 (2002).

8. Bullock, A.N. & Fersht, A.R. Rescuing the function of mutant p53. *Nat Rev Cancer* 1, 68-76 https://doi.org/10.1038/35094077 (2001).

9. Cho, Y., Gorina, S., Jeffrey, P.D. & Pavletich, N.P. Crystal structure of a p53 tumor suppressor-DNA complex: understanding tumorigenic mutations. *Science* 265, 346-55 https://doi.org/10.1126/science.8023157 (1994).

10. Joerger, A.C., Ang, H.C. & Fersht, A.R. Structural basis for understanding oncogenic p53 mutations and designing rescue drugs. *Proc Natl Acad Sci U S A* 103, 15056-15061 https://doi.org/10.1073/pnas.0607286103 (2006).

11. Suad, O. et al. Structural basis of restoring sequence-specific DNA binding and transactivation to mutant p53 by suppressor mutations. *J Mol Biol* 385, 249-265 https://doi.org/10.1016/j.jmb.2008.10.063 (2009).

12. Chen, S. et al. Arsenic Trioxide Rescues Structural p53 Mutations through a Cryptic Allosteric Site. *Cancer Cell* 39, 225-239 https://doi.org/10.1016/j.ccell.2020.11.013 (2021).
